# Cyanidin‐3‐galactoside from *Aronia melanocarpa* ameliorates silica‐induced pulmonary fibrosis by modulating the TGF‐β/mTOR and NRF2/HO‐1 pathways

**DOI:** 10.1002/fsn3.2861

**Published:** 2022-03-30

**Authors:** Yanmin Cui, Jin Zhao, Jing Chen, Yanwen Kong, Mingyue Wang, Yan Ma, Xianjun Meng

**Affiliations:** ^1^ 98428 College of Food Science Shenyang Agricultural University Shenyang PR China; ^2^ 12402 Center of Experiment Teaching Shenyang Normal University Shenyang PR China

**Keywords:** antioxidation, Cyanidin‐3‐galactoside, pulmonary fibrosis, silica particles

## Abstract

Cyanidin‐3‐galactoside (C3G), the most abundant anthocyanin in *Aronia melanocarp*a, has many beneficial health effects, such as antioxidation. C3G was extracted from *A. melanocarpa* and applied (100, 200, and 400 mg/kg body weight) to 50‐μl silica particles (SP) solution‐exposed mice to research its antifibrotic properties using histological analysis, hydroxyproline assay, quantitative real‐time polymerase chain reaction, and western blot analysis. The results showed that C3G treatment significantly ameliorated pulmonary fibrosis and cell infiltration into the lungs of mice. It also relieved SP‐induced epithelial–mesenchymal transition (EMT), 400 mg/kg C3G treatment increasing epithelial‐cadherin mRNA expression and decreasing α‐smooth muscle actin mRNA expression to the level of that in the control group. Western blot analysis showed that exposure to SP increased the production of transforming growth factor‐β1 (TGF‐β1) and phosphorylated mammalian target of rapamycin (mTOR) by 4.71‐ and 4.15‐fold, respectively, in the lungs of mice, which were significantly inhibited by C3G treatment. Moreover, 400 mg/kg C3G treatment up‐regulated two important antioxidant mediators, nuclear factor erythroid‐2‐related factor 2 (NRF2; 4.91‐fold) and heme oxygenase‐1 (HO‐1; 4.81‐fold). The mechanism study indicated that C3G might inhibit the TGF‐β/mTOR signaling via the NRF2/HO‐1 pathway and that SP‐induced pulmonary EMT was ameliorated by inhibiting the TGF‐β/mTOR signaling pathway. Our findings could provide new avenues for C3G as a functional food for preventing or mediating the progression of SP‐induced pulmonary fibrosis.

## Graphical abstract

Cyanidin‐3‐galactoside (C3G), the most abundant anthocyanin in *Aronia melanocarpa*, has many beneficial health effects, such as antioxidation. The mechanism study indicated that C3G might inhibit the transforming growth factor‐β (TGF‐β)/mammalian target of rapamycin (mTOR) signaling via the nuclear factor erythroid‐2‐related factor 2 (NRF2)/heme oxygenase‐1 (HO‐1) pathway and that silica particles (SP)‐induced pulmonary epithelial–mesenchymal transition (EMT) was ameliorated by inhibiting the TGF‐β/mTOR signaling pathway. The results indicate that C3G can be used as a complementary functional food to modulate pulmonary fibrosis caused by silica particles.

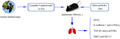

## 1 INTRODUCTION

As a natural anthocyanin, cyanidin‐3‐galactoside (C3G) is widely found in plants and has many beneficial health effects, such as antioxidation, regulating fat generation, and improving spatial memory (Adisakwattana et al., 2009; Bellocco et al., 2016; Lim et al., 2019; Long et al., 2014). *Aronia melanocarpa* or black chokeberry, native to North America and widely cultivated in countries including China, is known to be a rich source of anthocyanins, containing C3G as the most abundant anthocyanin (Chrubasik et al., 2010). The lung is a cavity organ directly exposed to the external environment, which is vulnerable to oxidative stress damage (Sunil et al., 2020). However, the protective effect of C3G on the lung is less studied, and its function and mechanism remain still unclear.

Industrialization increases workers’ exposure to inhalable particles, mostly silica particles (SP). Silicosis is a progressive occupational lung disease caused by long‐term inhalation of SP (Leung et al., 2012), remaining a worldwide burden due to poor surveillance (Steenland & Ward, 2014). Excessive extracellular matrix (ECM) protein deposition is the primary pathological characteristic (Lian et al., 2017), and many studies have demonstrated that ECM contains the major fibrillar collagens (collagen 1, 3, and 4) and fibronectin (Leppert et al., 2004). Epithelial–mesenchymal transition (EMT), a source of myofibroblasts, is increasingly considered a contributor to tissue fibrosis (Lamouille et al., 2014). Transforming growth factor‐β1 (TGF‐β1) is a potent inducer of EMT (Smith et al., 2015), and mammalian target of rapamycin (mTOR) plays a role in TGF‐β1‐induced EMT (Lamouille & Derynck, 2011). In addition, oxidative stress is involved in EMT induction by activating TGF‐β expression in the process of organ fibrosis (Cheresh et al., 2013b).

Our previous studies have attested the potently positive effects of anthocyanins on SP‐exposed mouse lungs (Zhao et al., 2018, 2019, 2020). Therefore, this study explored whether C3G from black chokeberry can alleviate SP‐induced pulmonary fibrosis in C57BL/6 mice.

## 2 MATERIALS AND METHODS

### 2.1 Preparation of C3G

The previously extracted C3G (Cui et al., 2021) was used in this study. We harvested black chokeberry “Fu Kangyuan No.1” from Liaoning FuKangyuan Black Chokeberry Technology Co., Ltd. and transported it at 4°C to Shenyang Agriculture University. Black chokeberry (500 g) was homogenized and extracted for 90 min in an ultrasonic bath (45°C, 500 W) with 0.1% HCl‐acidified ethanol at 1:5 (w/v) ratio. The extraction solution underwent vacuum filtration to remove the dross and rotary evaporation to remove the alcohol. It was then purified through D101 absorbent resins (Beijing Solarbio Science and Technology Co., Ltd.). To further isolate and purify the C3G, an LC‐3000 semi‐preparative HPLC (Beijing Tong Heng Innovation Technology Co., Ltd.) equipped with a Dikma Platisil C18 (300 × 10 mm, 10 μm) column and a G4212B diode‐array detector (Agilent Technologies) was used. Finally, the extract was freeze‐dried into powder and the purity of the powder was calculated (98.28%) by comparing its chromatogram with the chromatogram of a C3G standard using UPLC (H‐Class, Waters Corp.), as described in our previous paper (Cui et al., 2021), and then the powder was stored at −20°C until further use.

### 2.2 Total antioxidant capacity assessment

The antioxidant capacity of the C3G powder and vitamin C (VC) were estimated using T‐AOC, ABTS, and FRAP kits, respectively, as described by Meng et al. (2019).

#### 2.2.1 T‐AOC assay

The antioxidant molecules are able to reduce Fe^3+^ to Fe^2+^, forming a stable complex with phenanthroline, whose antioxidant capacity can be evaluated by colorimetry. A T‐AOC kit (Nanjing Jiancheng Bioengineering Institute) was used to determine the total antioxidant capacity of C3G and VC, and the experiment was performed in accordance with the manufacturer's instructions. The detection wavelength was 520 nm using an ELISA microplate reader, and one T‐AOC unit (U) was defined as a 0.01 increase in absorbance per milliliter of the reaction system of the sample to be tested at 37°C per minute.

#### 2.2.2 ABTS assay

ABTS is oxidized to green ABTS^+^ under the action of a proper oxidant and it will be inhibited by antioxidants. The total antioxidant capacity of the samples was calculated according to the absorbance of ABTS^+^ at 734 nm. An ABTS kit (Nanjing Jiancheng Bioengineering Institute) was used to detect the total antioxidant capacity of C3G and VC. The sample (10 mg in 2‐ml methanol) was prepared and Trolox was selected as the standard solution. The antioxidant capacity was calculated according to the absorbance of ABTS^+^ at 734 nm and the linear Trolox calibration curve. The results expressed as Trolox equivalents/mM.

#### 2.2.3 FRAP assay

Under acidic conditions, antioxidants can reduce Fe^3+^‐TPTZ to produce blue Fe^2+^‐TPTZ, and the total antioxidant capacity can be calculated by measuring the absorbance at 593 nm. A FRAP kit (Nanjing Jiancheng Bioengineering Institute) was used to detect the total antioxidant capacity of C3G and VC. The antioxidant capacity was calculated according to the linear calibration curve of fresh FeSO_4_ solutions and expressed as FeSO_4_.7H_2_O equivalents/mM.

### 2.3 Animal treatment

Male C57BL/6 mice (6–8 weeks old, weight 18–22 g) were purchased from Liaoning Changsheng Biotechnology Co. Ltd. and bred in a pathogen‐free environment at 23 ± 1°C with a 12/12‐h light/dark cycle, having free access to water and feed. All experiments were performed according to the protocols approved by Shenyang Agricultural University Institutional Research Board (License No. SYXK<Liao>2011–0001).

The pulmonary fibrosis mouse model was developed in accordance with previously published methods (Du et al., 2019; Zhao et al., 2019). The SP were purchased from SILICA Corp. with a mean diameter of 1.5 μm. After 1 week of acclimation, mice were randomly divided into five groups, each with eight mice. Mice in the control group (saline) were intratracheally instilled with 50‐μl saline solution. Mice in the model group (silica) were intratracheally instilled with 50‐μl SP solution (10 mg/ml SP in saline) and mice in the experimental groups (silica+C3G100, silica+C3G200, and silica+C3G400) were administered 50‐μl SP solution (10 mg/ml SP in saline) on day 0. The mice in the experimental group were rendered 50 μl C3G by gavage (100, 200, and 400 mg/kg body weight in distilled water, respectively) daily from day 0, while mice in the control and model groups were correspondingly administered saline solution. After 56 days, the animals were sacrificed with Zoetic–Rompuy mixture by intraperitoneal infusion. The lungs were excised, snap‐frozen in liquid nitrogen, and stored at −80°C for later use.

### 2.4 Histological analysis

The lung tissue samples were immersed in paraffin which was segmented at a thickness of 5 μm to perform histological analysis. The results were visualized using a DP73 light microscope (Olympus).

#### 2.4.1 Hematoxylin and eosin (H&E) staining

To observe the basic pathological morphology of the lung tissue structure, sample sections were stained with hematoxylin and eosin (H&E) after dewaxed using xylene. Section dehydration, clarifying, and sealing were then performed using ethanol, xylene, and neutral gum, respectively. Finally, the staining results were observed under the microscope.

#### 2.4.2 Immunohistochemistry staining

Immunohistochemistry (IHC) staining was performed to observe the distribution of collagen 1. After the antigen of the sections was retrieved in citrate buffer, the sections were then incubated with collagen 1 primary antibody (Affinity Biosciences) overnight at 4°C and with a horseradish peroxidase‐conjugated secondary antibody for 60 min at 37°C, followed by 3,3‐diaminobenzidine color development (Fuzhou Maixin Biotechnology Development Co. Ltd.).

#### 2.4.3 Masson staining

Masson staining was used to determine the degree of fibrosis based on a method described by Du et al. (2019). The paraffin sections of the samples were dewaxed, washed, stained with hematoxylin for 6 min, and then re‐stained with acid ponceau solution for 1 min. After being embathed in 0.2% glacial acetic acid, the sections were treated with 1% phosphomolybdic acid for 5 min, and finally, stained with aniline blue for 5 min and dehydrated.

### 2.5 Hydroxyproline assay

Hydroxyproline (HYP) content was determined using an HYP kit (Nanjing Jian Cheng Institute) to estimate collagen deposition. The left lung tissue (100 mg wet weight) was accurately weighed, mixed with 1‐ml hydrolysate, and placed in a boiling water bath for 20 min, which was followed by treatment with the HYP kit based on the manufacturer's instructions. The results were expressed as HYP (μg)/wet lung (mg).

### 2.6 Quantitative real‐time polymerase chain reaction

Total RNA was extracted from lung tissues using a Total RNA Extraction Kit (Wanlei Biotechnology Co., Ltd.). RNA was transcribed into cDNA using the Revertaid First Strand cDNA Synthesis Kit (Thermo Fisher Scientific Inc.). Reactions were performed in a Light Cycler 96 instrument (Roche) using UltraSYBR Mixture as the fluorescent dye. Reduced glyceraldehyde‐phosphate dehydrogenase (GAPDH) was used as the internal control, and data were analyzed according to the expression of 2^−ΔΔ^ *^Ct^* (Wang et al., 2019). The primer sequences were as follows: epithelial (E)‐cadherin (F: ATCCTCGCCCTGATT, R: ACCACCGTTCTCCTCCGTA), α‐smooth muscle actin (α‐SMA) (F: CGGGCTTTGCTGGTGATG, R: GGTCAGGATCCCTCTCTTGCT), and GAPDH (F: GTCTTCACCACCATGGAGAAG, R: GTTGTCATGGATGACCTTGGC).

### 2.7 Western blot analysis

We collected sample lungs and lysed them for 5 min in buffer with 1% proteinase inhibitors on ice, and then a BCA assay kit (Wanlei Biological Technology Co., Ltd.) was used to determine protein concentrations in the supernatant. The protein (40 μg) was subjected to sodium dodecyl sulfate–polyacrylamide gel electrophoresis and transferred onto polyvinylidene fluoride (PVDF) membranes, after which, PVDF membranes were blocked with 5% nonfat milk containing Tris‐buffered saline with Tween for 1 hr at room temperature. The membranes were then incubated with primary antibodies against TGF‐β1 (Affinity Biosciences) and phosphorylated (p)‐mTOR (Affinity Biosciences) overnight at 4°C and incubated with secondary antibody for 60 min at room temperature. β‐actin was used as an internal control that showed no differences between test groups. Luminescence signals were detected with an enhanced chemiluminescence system (Wanlei Biotechnology Co., Ltd.), and Gel‐Pro‐Analyzer software version 4.0 (Media Cybernetics Inc.) was used to analyze signal strength.

### 2.8 Statistical analysis

Data were expressed as means ±standard deviations and were analyzed using SPSS Statistics v. 22.0 software (IBM Corp.). Comparisons between differently treated groups were performed using the Student–Newman–Keuls test following the one‐way analysis of variance. A value of *p* < .05 was considered statistically significant. Graphing was completed using Origin Pro 9.1 (Originlab Corporation), Excel software (Version 2016; Microsoft Corp.), and Adobe Photoshop CC 14.0 (Adobe Photoshop).

## 3 RESULTS

### 3.1 Determination of antioxidant capacity of C3G

**FIGURE 1:**
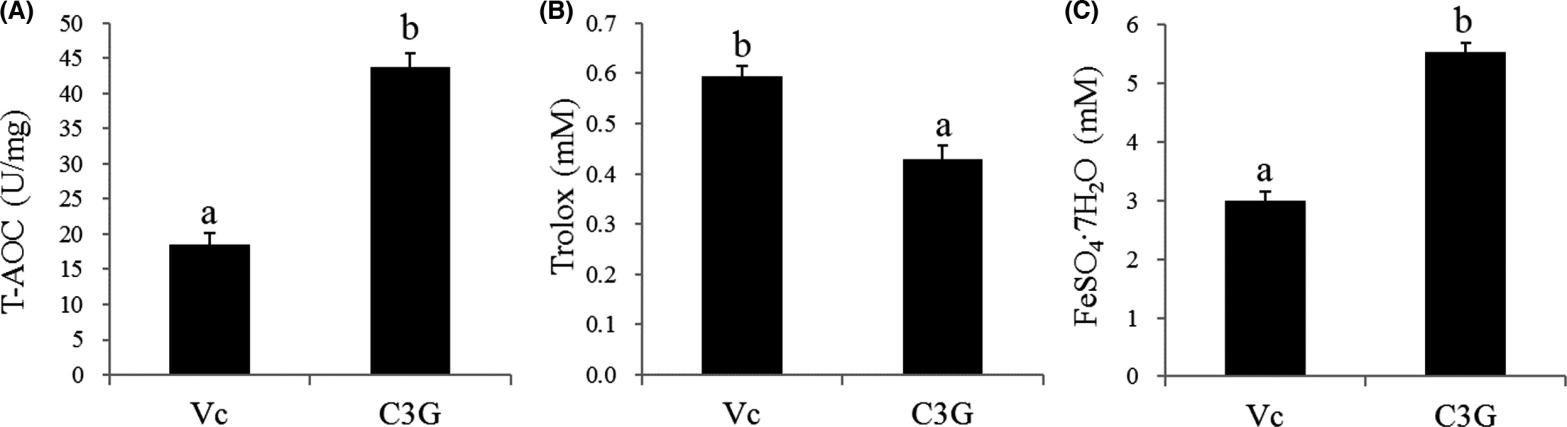
Antioxidant activities of cyanidin‐3‐galactoside (C3G) and vitamin C (VC), using (A) T‐AOC, (B) ABTS, and (C) FRAP assays. Results are expressed as the mean ± *SD* (*n* = 3). Different lowercase letters indicate statistically significant differences (*p* < .05)

In the T‐AOC assay, the antioxidant activity of C3G was 43.76 U/mg, which was significantly higher (*p* < .05; by 2.36‐fold) than that (18.49 U/mg) of VC (Figure 1A). Although the ABTS^+^ scavenging ability of C3G was 0.43 mM and VC had stronger activity (0.59 mM; Figure 1B), Figure 1C showed that the FRAP value of C3G was higher by 1.84‐fold than that of VC. The findings showed that C3G had the potent antioxidative capability.

### 3.2 C3G alleviates SP‐induced pulmonary fibrosis response

**FIGURE 2:**
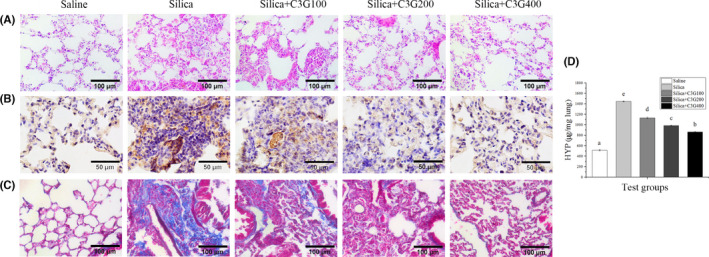
Cyanidin‐3‐galactoside (C3G) alleviates the pulmonary fibrosis response induced by silica particles in C57BL/6 mice. (A) Hematoxylin and eosin, (B) collagen 1 immunohistochemistry, and (C) Masson staining of lung tissue sections. Scale bars: 100, 50, and 100 μm (*n* = 4–5). (D) Hydroxyproline (HYP) assay of lung tissues. Results are expressed as the mean ± *SD* (*n* = 3). Different lowercase letters indicate statistically significant differences (*p* < .05)

Lung tissue is mainly composed of collagen and elastin fibers, which is essential for maintaining the elasticity required for the normal lung volume. The excessive deposition of ECM proteins results in decreased effective elasticity, making breathing difficult (Karkale et al., 2018). After SP‐exposed mice were treated with C3G (100, 200, and 400 mg/kg body weight) for 56 consecutive days, H&E staining exhibited that C3G treatment decreased the sizes of SP‐induced cell nodules (Figure 2A). IHC staining for collagen 1, an ECM marker, was utilized to determine the effect of C3G on SP‐induced pulmonary fibrosis. C3G treatment reduced the level of collagen 1 compared with that in the SP group (Figure 2B). Masson staining showed that SP‐induced fibrosis was alleviated by C3G treatment, which was positively correlated with the dose (Figure 2C). HYP is a key amino acid precursor for collagen synthesis in the fibrotic lesions of mice (Liu et al., 2017), and its content was determined to further assess the degree of fibrosis. As shown in Figure 2D, compared to the control group, SP treatment increased the amount of HYP by 2.81‐fold, but treatment with 100, 200, and 400 mg/kg C3G alleviated this increase to 2.2‐, 1.92‐, and 1.63‐fold, respectively. These results indicated that C3G treatment could effectively alleviate SP‐induced pulmonary fibrosis.

### 3.3 C3G ameliorates SP‐induced pulmonary EMT

**FIGURE 3:**
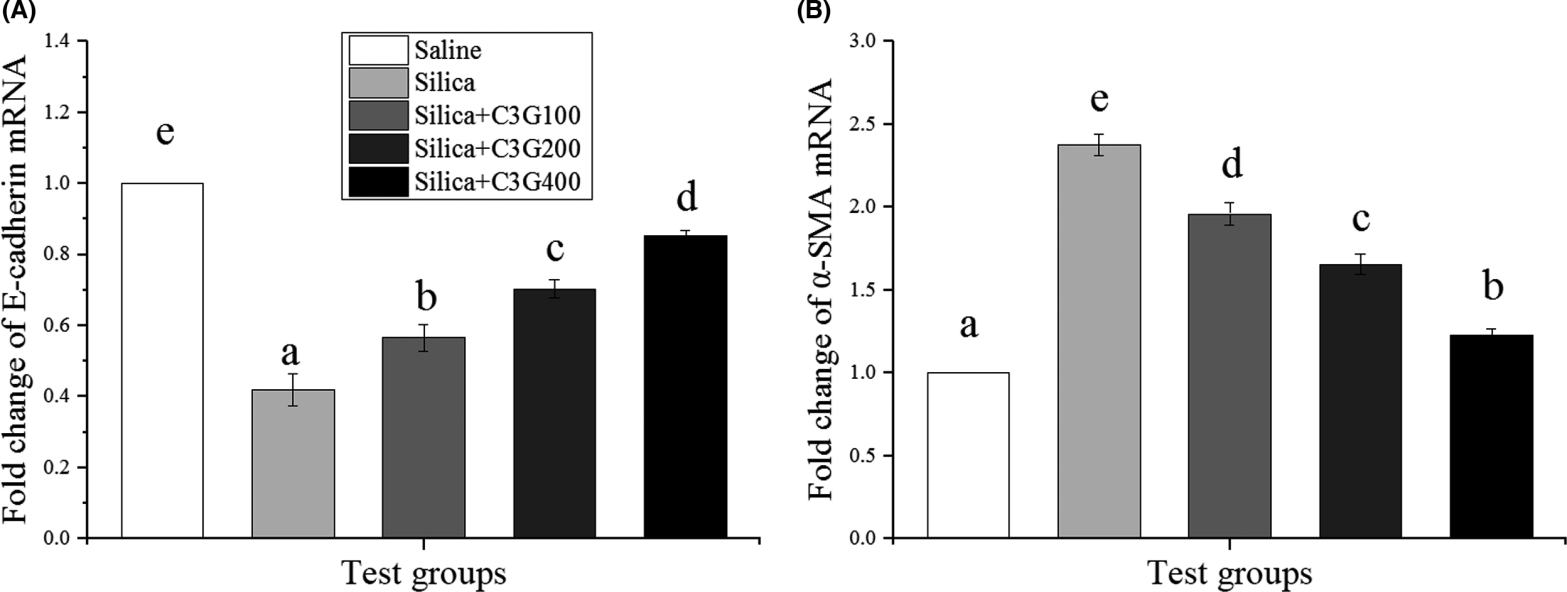
Cyanidin‐3‐galactoside (C3G) ameliorates silica particles‐induced pulmonary epithelial–mesenchymal transition. Quantitative real‐time polymerase chain reaction was used to determine the fold change of (A) epithelial (E)‐cadherin mRNA and (B) α‐smooth muscle actin (α‐SMA) mRNA expression in different groups. Results are expressed as the mean ± *SD* (*n* = 3). Different lowercase letters indicate statistical differences (*p* < .05)

Epithelial–mesenchymal transition, during which epithelial cells lose several epithelial characteristics, such as E‐cadherin, and acquire typical properties of mesenchymal cells, such as α‐SMA, has been considered to play a crucial part in the development of fibrosis (Liu, 2004; Liu et al., 2017). As shown in Figure 3A, SP treatment significantly (*p* < .05) decreased the mRNA expression of E‐cadherin to 58% of that in the control group, while treatment with 100, 200, and 400 mg/kg C3G increased the SP‐induced mRNA expression of E‐cadherin to 57%, 70%, and 85%, respectively. In comparison, SP treatment increased α‐SMA mRNA expression by 2.38‐fold compared to that in the control group, but treatment with 100 and 200 mg/kg C3G lowered the expression to 1.96‐ and 1.66‐fold, respectively, and 400 mg/kg C3G treatment lowered the expression almost to the level observed in the control group (Figure 3B). These results confirmed the anti‐EMT capability of C3G.

### 3.4 C3G ameliorates SP‐induced pulmonary EMT by inhibiting the TGF‐β/mTOR signaling

**FIGURE 4:**
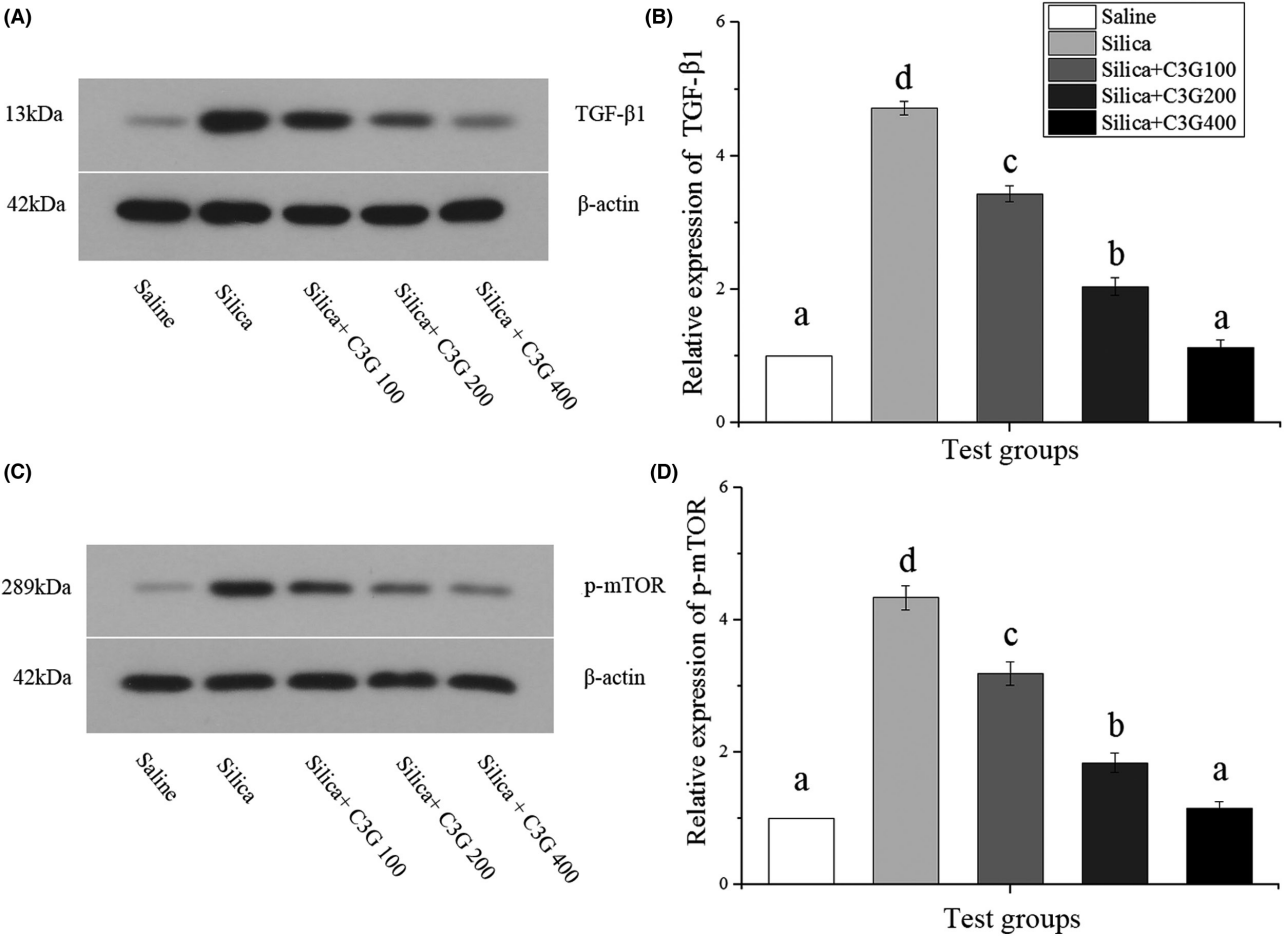
Cyanidin‐3‐galactoside (C3G) ameliorates silica particles‐induced pulmonary epithelial–mesenchymal transition by inhibiting transforming growth factor‐β1 (TGF‐β1)/mammalian target of rapamycin (mTOR) signaling. Western blot analysis was used to determine protein expression in different groups. (A, B) Protein expression of TGF‐β1. (C, D) Protein expression of phosphorylated (p)‐mTOR. Protein expression was normalized to that of β‐actin. Results are expressed as the mean ± *SD* (*n* = 3). Different lowercase letters indicate statistical differences (*p* < .05)

Transforming growth factor‐β mRNA and/or protein outflow is expanded in fibrotic diseases in almost every organ system of humans and test creature models (Cheresh et al., 2013a). mTOR belongs to the phosphatidylinositol kinase‐associated kinase family and plays a vital part in many cellular activities (Bai et al., 2010). To investigate the molecular mechanisms of C3G‐mediated EMT attenuation, we used western blot to analyze the lung tissue samples of mice from different treatment groups. We found an increase in TGF‐β1 expression by 4.71‐fold after SP treatment, which was significantly ameliorated by 100 and 200 mg/kg C3G treatment to 3.43‐ and 2.04‐fold, respectively, and 400 mg/kg C3G treatment ameliorated the expression to the level observed in the control group (Figure 4A,B). As shown in Figure 4C,D, treatment with SP significantly (*p* < .05) increased p‐mTOR expression by 4.15‐fold compared to that in the control group, while 100 and 200 mg/kg C3G treatment decreased SP‐induced p‐mTOR expression to 3.39‐ and 2.00‐fold, respectively, and 400 mg/kg C3G treatment ameliorated the expression to a normal level.

### 3.5 C3G inhibits the TGF‐β/mTOR signaling via the NRF2/HO‐1 pathway

**FIGURE 5:**
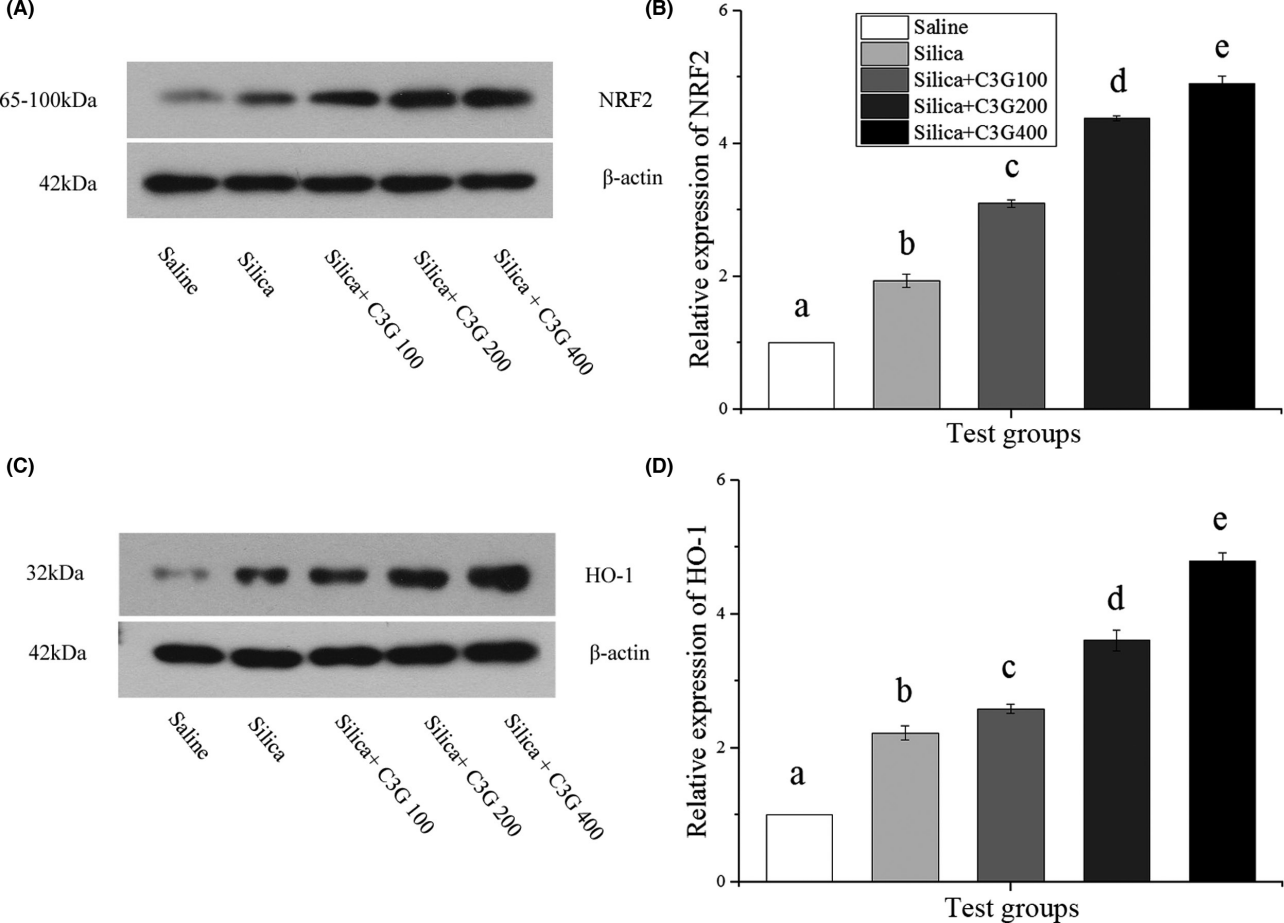
Cyanidin‐3‐galactoside (C3G) inhibits transforming growth factor‐β1 (TGF‐β1)/mammalian target of rapamycin (mTOR) signaling via the nuclear factor erythroid‐2‐related factor 2 (NRF2)/heme oxygenase‐1 (HO‐1) pathway. Western blot analysis was used to determine protein expression in different groups. (A, B) Protein expression of NRF2. (C, D) Protein expression of HO‐1. Protein expression was normalized to that of β‐actin. Results are expressed as the mean ± *SD* (*n* = 3). Different lowercase letters indicate statistical differences (*p* < .05)

Persistent oxidative stress has been shown to promote SP‐induced pulmonary fibrosis to some extent (Mascarenhas et al., 2018). Antioxidant mediators are significant in neutralizing excessive oxidative stress (Reuter et al., 2010). Nuclear factor erythroid‐2‐related factor 2 (NRF2) is considered the “master regulator” of oxidative stress (Hybertson et al., 2011). We examined whether NRF2 and heme oxygenase‐1 (HO‐1), two vital antioxidant mediators, play protective roles in the effects of C3G against SP‐induced oxidative stress. As shown in Figure 5A,B, the NRF2 level increased by 1.93‐fold in the mouse lungs after SP instillation compared to that in the control group, while 100, 200, and 400 mg/kg C3G treatment further up‐regulated this by 3.10‐, 3.54‐, and 4.91‐fold, respectively. Figure 5C,D showed that SP treatment increased the HO‐1 expression level by 2.22‐fold compared with that in the control group, whereas 100, 200, and 400 mg/kg C3G treatment significantly (*p* < .05) up‐regulated the HO‐1 expression level by 2.59‐, 3.61‐, and 4.81‐fold, respectively. These findings implied the potential of C3G treatment for increasing resistance to oxidative stress.

## 4 DISCUSSION

Silicosis is the most prominent occupational lung disease globally, particularly in developing countries, which is a major health problem affecting over 35 million workers worldwide in coal mining and other dusty industries, such as sand blasting, construction, and metallurgy (Chen et al., 2018). Here, we investigated the effects of C3G from *A. melanocarpa* on SP‐induced pulmonary fibrosis in mice and its cellular mechanism. The results showed that C3G might play a positive role in the lungs by alleviating EMT, regulating the TGF‐β/mTOR pathway, and reducing oxidative stress through the NRF2/HO‐1 pathway.

In this study, we found that C3G treatment alleviated cell nodule formation and collagen deposition caused by SP in the lungs of mice using H&E, IHC, and Masson staining (Figure 2A,C). The increased HYP content is reckoned as a biomarker of the aggravation of pulmonary fibrosis (Desaki et al., 2002). The results showed that compared with that in the control group, the lung HYP level in the model group increased, indicating that C3G treatment attenuated the increase of SP‐induced HYP in a dose‐dependent manner (Figure 2D), which coincide with the results described by Zhao et al. (2019). Therefore, C3G treatment can effectively alleviate SP‐induced pulmonary fibrosis.

Transforming growth factor‐β, existing as TGF‐β 1, 2, and 3, exerts important effects in ECM deposition by regulating pivotal biological responses like EMT (Cheresh et al., 2013a). As expected, we found evidence supporting EMT in the model group and alleviated EMT in the experimental group (Figure 3). TGF‐β1, one of the many cytokines that regulate EMT, is the potent inducer of EMT in various organ systems that functions through the activation of downstream Smad and non‐Smad signaling pathways (Ji et al., 2016; Lamouille & Derynck, 2011). Among the non‐Smad pathways, evidence continues to accumulate showing that the phosphoinositide 3–kinase–protein kinase B–mTOR axis plays an important role in TGF‐β‐induced EMT (Lamouille & Derynck, 2011). Relevant evidence suggests that mTOR inhibition effectively inhibits the fibrosis process in both experimental models of radiation‐ and bleomycin‐induced pulmonary fibrosis (Helal & Said, 2019). As indicated in Figure 4, C3g treatment significantly (*p* < .05) induced the down‐regulation of TGF‐β1 and p‐mTOR proteins compared to expression in the model group. Therefore, C3G treatment might ameliorate SP‐induced pulmonary EMT by inhibiting TGF‐β/mTOR signaling.

Oxidative stress is a crucial molecular mechanism of fibrosis in various organs, including the lungs (Cheresh et al., 2013a). Lungs are more susceptible to oxidative stress than other organs due to exposure to the highest levels of oxygen (Cheresh et al., 2013a). Normally, Keap1 binds to NRF2, leading to the proteasomal degradation of NRF2 through ubiquitination (David et al., 2017). When the oxidation state is stronger than the reduction state, resulting in a disruption of the balance, NRF2 plays a role by translocating from the cytoplasm to the nucleus and combining with antioxidant response elements, to up‐regulate the transcription of genes involved in metabolism, protein homeostasis, and the redox balance (Patinen et al., 2019). NRF2 also augments the activity of HO‐1 (Sugumar et al., 2020). Oxidative stress can activate latent TGF‐β, and TGF‐β stimulates oxidative stress, thus setting up a vicious cycle (Cheresh et al., 2013a). In this study, SP reduced the expression of NRF2 and HO‐1 in the model group compared to levels in the control group, and C3G further up‐regulated their expression with an increase in its concentration (Figure 5). Therefore, C3G treatment might inhibit TGF‐β/mTOR signaling by the NRF2/HO‐1 pathway to alleviate SP‐induced pulmonary fibrosis. Similar to the results of the study of David et al. (2017), the results obtained here confirmed that NRF2 might be a novel target for the treatment of pulmonary fibrosis.

Although silicosis is characterized by pulmonary fibrosis caused by long‐term inhalation of SP, its pathogenesis is still unknown and there is no specific treatment (Zhu et al., 2020). Many phytoconstituents, such as resveratrol, curcumin, baicalein A, and berberine, have been found to effectively inhibit organ fibrosis (Bahri et al., 2017). C3G is the most abundant anthocyanin in *A. melanocarpa*, which exerts many healthy effects, and its antioxidant capacity is directly relevant to its flavylium cation (AH+) structure (Tena et al., 2020). As shown in Figure 1, C3g has a strong antioxidant capacity. Many previous studies have shown the importance of oxidative stress in organ fibrosis. However, whereas all organs express a diversity of antioxidants to combat oxidative stress, a large number of antioxidant trials have not shown improvement in patients with fibrotic disease (Cheresh et al., 2013a). Although the antioxidant activity of polyphenols including anthocyanin may have crucial effects on alleviating oxidative stress, their actual effect at the cellular level could be more complex (Tsao, 2010). There is a perspective that polyphenols and their metabolites play a regulatory role in cells by acting on protein kinase and lipid kinase signaling pathways in vivo (Williams et al., 2004). Therefore, for C3G approval as an effective biomolecule against fibrosis, it is necessary to continue the investigation of its effect as an antioxidant or regulator of cellular metabolism. Furthermore, the antifibrotic effect of bone morphogenetic protein‐7 in the prevention group was more effective than that in the treatment group (Liang et al., 2016). Thus, a direction for future research related to this work is to study the preventive effect and molecular mechanism of C3G.

## 5 CONCLUSION

In this study, the effect of C3G treatment was studied on SP‐induced pulmonary fibrosis using an SP‐exposed mouse model. The results demonstrated that C3G treatment significantly ameliorated pulmonary fibrosis in mice with an increase in its concentration. Treatment with C3G inhibited EMT via the TGF‐β/mTOR pathways and reduced the oxidative stress via the NRF2/HO‐1 pathway, and NRF2/HO‐1 pathway inhibited the TGF‐β/mTOR signaling. Based on these results, we believe that C3G could be used as a novel food supplement and play an important role in the prevention or treatment of pulmonary fibrosis caused by SP.

## CONFLICT OF INTEREST

The authors declare no conflict of interest.

## Data Availability

The data that support the findings of this study are available from the corresponding author upon reasonable request.
